# TRANSITION AND STABILITY OF PLACE AND AGING IN LATER LIFE: HOUSING AND NEIGHBORHOOD CONTEXTS

**DOI:** 10.1093/geroni/igad104.0734

**Published:** 2023-12-21

**Authors:** Frank Oswald, Steven Schmidt, Malcolm Cutchin

## Abstract

Transition and stability in housing and neighborhood contexts reflect both, a central field of research and policies on place and aging for health and well-being in later life. Studies have demonstrated effects of improving the physical environment, particularly with respect to specific needs, such as for people living with dementia. Moreover, relationships between perceived housing and health and well-being on the one hand and life transitions and health and well-being on the other hand have been addressed. However, research on complex interactions of place and transitions in later life is scarce. This symposium aims to address the dynamic relationship between transition and stability of place and aging in later life in relation to indicators of good ageing. The first contribution by Eriksson and colleagues presents quantitative results on the relationships between perceived housing and changes in health and well-being over time among community-dwelling Swedish older adults. Second, Kylén and colleagues present quantitative data describing which housing preferences are most important when considering relocation in older age. The third contribution by Slaug and colleagues uses a quantitative approach to qualitative interview data from Sweden and Germany, analyzing sequences of and relationships between life transitions over 10 years. Fourth, Chaudhury and colleagues show data that aims to identify spatial and temporal patterns in activities undertaken outside home by people living with dementia, and ways in which the neighborhood-built environment affects their outdoor mobility and social participation. Finally, Malcom P. Cutchin will serve as the session’s discussant.

